# Time-Lapse Helical X-ray Computed Tomography (CT) Study of Tensile Fatigue Damage Formation in Composites for Wind Turbine Blades

**DOI:** 10.3390/ma11112340

**Published:** 2018-11-21

**Authors:** Ying Wang, Lars P. Mikkelsen, Grzegorz Pyka, Philip J. Withers

**Affiliations:** 1 Henry Moseley X-ray Imaging Facility, Henry Royce Institute for Advanced Materials, School of Materials, University of Manchester, M13 9PL Manchester, UK; ying.wang-4@manchester.ac.uk; 2 Composite Mechanics and Structures, Department of Wind Energy, Technical University of Denmark, DK-4000 Roskilde, Denmark; lapm@dtu.dk; 3 Thermo Fisher Scientific Czech Republic, 67200 Brno, Czech Republic; grzegorz.pyka@thermofisher.com

**Keywords:** helical CT, contrast agent, high cycle fatigue (HCF), fibre break, fibre tows

## Abstract

Understanding the fatigue damage mechanisms in composite materials is of great importance in the wind turbine industry because of the very large number of loading cycles rotor blades undergo during their service life. In this paper, the fatigue damage mechanisms of a non-crimp unidirectional (UD) glass fibre reinforced polymer (GFRP) used in wind turbine blades are characterised by time-lapse ex-situ helical X-ray computed tomography (CT) at different stages through its fatigue life. Our observations validate the hypothesis that off-axis cracking in secondary oriented fibre bundles, the so-called backing bundles, are directly related to fibre fractures in the UD bundles. Using helical X-ray CT we are able to follow the fatigue damage evolution in the composite over a length of 20 mm in the UD fibre direction using a voxel size of (2.75 µm)^3^. A staining approach was used to enhance the detectability of the narrow off-axis matrix and interface cracks, partly closed fibre fractures and thin longitudinal splits. Instead of being evenly distributed, fibre fractures in the UD bundles nucleate and propagate locally where backing bundles cross-over, or where stitching threads cross-over. In addition, UD fibre fractures can also be initiated by the presence of extensive debonding and longitudinal splitting, which were found to develop from debonding of the stitching threads near surface. The splits lower the lateral constraint of the originally closely packed UD fibres, which could potentially make the composite susceptible to compressive loads as well as the environment in service. The results here indicate that further research into the better design of the positioning of stitching threads, and backing fibre cross-over regions is required, as well as new approaches to control the positions of UD fibres.

## 1. Introduction

Composite wind turbine rotor blades undergo a very large number of fatigue loading cycles during service [1]. As a result, their fatigue performance is a major design factor as high cycle fatigue (HCF) damage can result in unexpected catastrophic failure. Consequently, it is important to understand damage evolution during fatigue, the key damage mechanisms and the interaction between them for the unidirectional (UD) composites employed in wind turbines. 

X-ray computed tomography (CT), which has been applied increasingly to materials characterisation [2,3], is superior to most non-destructive techniques in that three-dimensional (3D) information can be obtained non-destructively at a high spatial resolution. Unlike fatigue crack initiation and propagation in homogeneous materials, various damage mechanisms occur cooperatively in composites under cyclic loading, including fibre fracture, matrix cracking, debonding and delamination [4]. Establishing a time evolving 3D map of the complex fatigue damage modes in relation to local microstructure will contribute to the establishment and validation of models of fatigue failure able to better predict the safe life of such composites.

As observed in a number of composite systems, fatigue damage originates from cracks within fibre bundles or individual fibre fractures [5,6,7,8], which are on the micron level in size. High-resolution X-ray CT is needed to visualise these features, but high resolution (small voxel size) often means a small field-of-view (FoV), usually much smaller than is sampled by mechanical tests and often shorter than is needed to statistically characterise the failure processes operative in composites unless a significant number of images are stitched together. The use of helical X-ray CT enables a significant length of the sample to be imaged in a single scan. Furthermore the helical method avoids ring artefacts and cone beam artefacts [9], and can lead to high quality images which can be important when imaging low contrast systems or trying to detect events near the spatial resolution limit of the instrument. Consequently, helical CT is well suited to the characterisation of unidirectionally reinforced fibre composites, allowing us to observe the overall damage distribution and to detect localised damage events such as individual fibre fractures. In addition, dye penetrant with high atomic number (e.g., zinc iodide) can be used as stains to improve the detectability of cracks by enhancing the contrast between damage and the bulk material [2,3]. Although the use of staining has limitations in that only cracks connected to the outer surface could be stained and that it could affect the growth of matrix crack/splitting under fatigue [10], Yu et al. [11] assessed the effect of four methods to increase detectability of cracks in X-ray CT imaging and suggested that staining was perhaps the most effective in terms of increasing the sensitivity of cracks to better than 1/10 the spatial resolution. 

With regard to reproducibility we have analysed three samples in this project using the strategy of combining helical imaging with staining for damage characterisation. The damage mechanisms observed are similar across all three samples (S1, S2 and S3). In reference [12], the effectiveness of this strategy and the distribution of UD fibre fractures were discussed based on two of these (S1 and S2) fatigued at maximum stress equal to 0.5% initial strain. The damage mechanisms were also found to be similar to those for unstained samples [8]. 

In this paper, the aim is to track the evolution, distribution and interaction between fatigue damage events and to relate these to the composite microstructure of the glass fibre reinforced polymer (GFRP) using time-lapse helical X-ray CT, and discussion focuses on one sample (S3) fatigued at maximum stress equalling 0.6% initial strain. 

## 2. Materials and Methods

**Figure 1:**
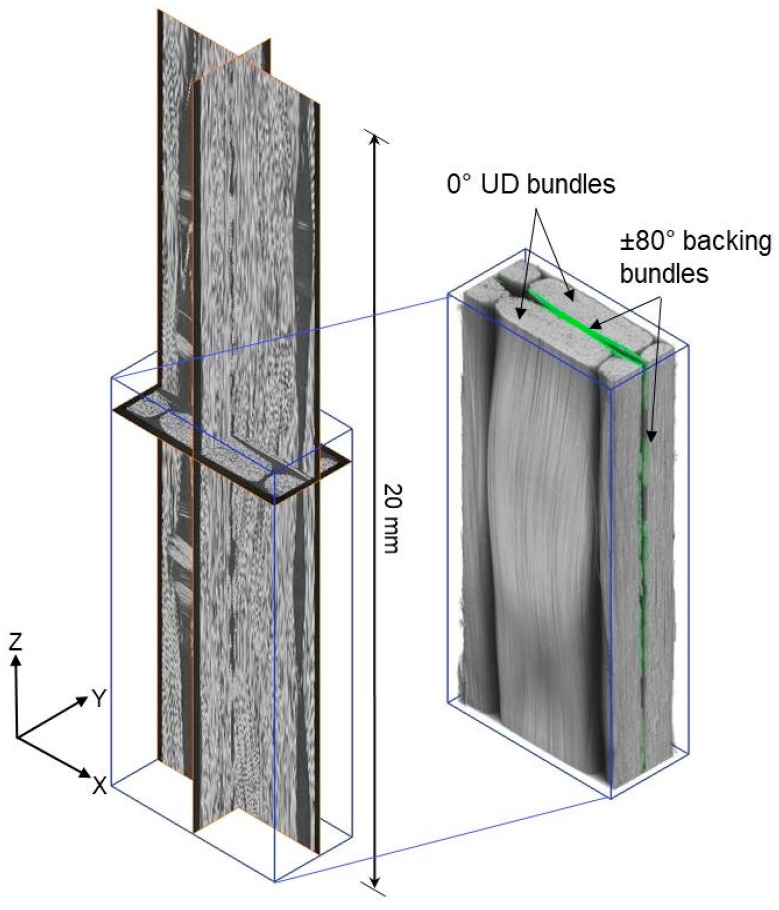
Orthogonal virtual X-ray CT sections (**left**) and a 3D volume rendering (**right**) showing the fibre architecture of the GFRP material where the ±80° backing fibre bundles are rendered in green.

**Figure 2:**
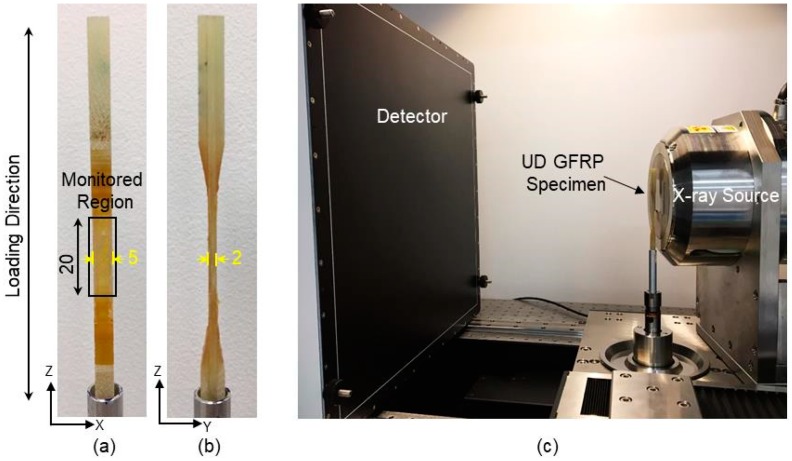
(**a**,**b**) Photographs illustrating the GFRP specimen geometry and the X-ray CT monitored region through interrupted fatigue test (dimensions in mm). (**c**) Photograph showing the helical X-ray CT imaging set-up in the ThermoFisher HeliScan Micro-CT scanner.

The composite material studied is a glass fibre/polyester composite system typically used in wind-turbine rotor blades. The GFRP sample has a lay-up of [0/b]_S_, where ‘0’ represents a 0° UD fibre layer and ‘b’ corresponds to a thin (~100–200 µm) ±80° backing fibre layer. Figure 1 shows the orthogonal virtual CT sections and a 3D rendered CT image of the fibre architecture. The composite panel was manufactured using vacuum assisted resin transfer moulding (VARTM). Specimens having dimensions of 2 mm × 5 mm × 110 mm were cut from the composite panel and GFRP tabs were added to the ends of the test-pieces (see Figure 2a,b). This miniaturised specimen geometry was used in order to obtain a high spatial resolution, given that the full specimen width should ideally lie within the FoV during the scan if the reconstruction algorithm is not to introduce artefacts [9].

Fatigue tests were performed on a hydraulic Instron 8802 (Norwood, MA, USA) mechanical testing machine under load control with a sinusoidal waveform and a stress ratio (R = σ_min_/σ_max_, where σ_min_ is the minimum stress and σ_max_ is the maximum stress) of 0.1 at a test frequency of 5 Hz. The maximum stress applied corresponds to 0.6% initial strain on the composite. The fatigue test was interrupted periodically with increasing numbers of fatigue cycles to monitor the damage evolution using ThermoFisher HeliScan (ThermoFisher Scientific, Brno, Czech Republic) helical X-ray CT scanner. The same sample was removed from the loading frame for CT inspection after 0 cycle (N_0_), 50,000 cycles (N_1_) and 500,000 cycles (N_2_) respectively. 

After each fatigue increment the sample was removed from the testing frame and stained in zinc iodide solution for 24 h before being imaged on the CT scanner. The zinc iodide solution was prepared following the method used by Nixon-Pearson et al. [13]. It should be noted that the specimen was stained in the absence of any load to open the cracks. Figure 2c shows the imaging set-up. The source voltage was set to 80 kV and filtered by 0.1 mm of stainless steel to remove the low energy X-rays. The exposure time for each projection (radiograph) was 0.52 s with around 20,000 projections acquired in all. The double-helix mode was used to allow reconstruction using filtered-back-projection algorithms. During the scan, the sample stage simultaneously rotates and translates vertically following a helical path with a pitch of ~7.8 mm. The scanned composite volume extends to ~20 mm in height and has a FoV height-to-width ratio of >3 at a pixel size of 2.75 µm, resulting in a total scan time of ~20 h and reconstruction time of ~3 h. High-resolution region-of-interest (RoI) scans at a pixel size of 1 µm were also taken after the time-lapse study to confirm the presence of unstained cracks.

## 3. Results and Discussion

**Figure 3:**
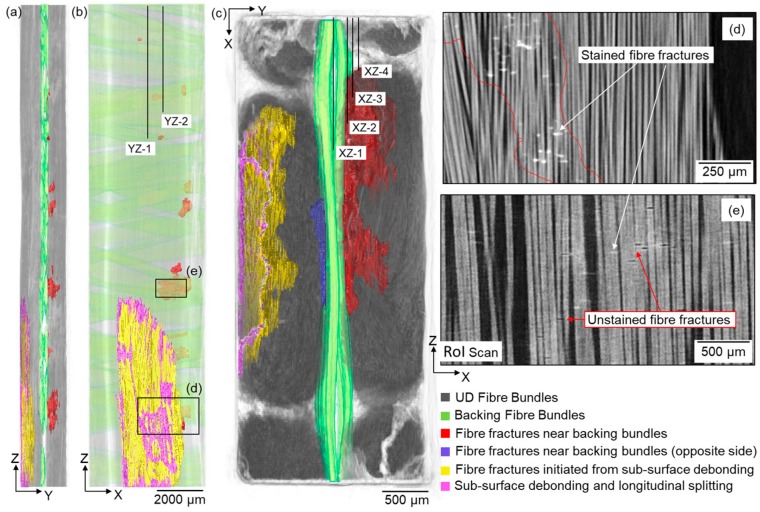
X-ray CT 3D volume rendering of the extracted damage after 500,000 cycles (N_2_), where the fibre fractures, longitudinal splits and debonding are visualised with respect to the UD and backing fibre bundles, (**a**) YZ view, (**b**) XZ view, (**c**) XY view (refer to Figure 2a,b for definition of the coordinate system). (**d**) Typical virtual X-ray CT XZ of the region marked in (**b**), illustrating the manual annotation of fibre fracture damage in the CT image. (**e**) High-resolution RoI X-ray CT virtual XZ section of the damage region marked in (**b**) where some fibre fractures were not stained.

Using traditional circular X-ray CT, it is challenging and time-consuming to locate the RoI to perform time-lapse tracking of the damage evolution due to the limited FoV. Based on previous studies of this material by Jespersen et al. [8,14,15], it is expected that fatigue damage is most likely to initiate from regions where the backing bundles cross-over; however, it is important to confirm whether this is generally true for this material system or whether there are other factors that might localise fatigue damage. Owing to the extended length that can be viewed at high resolution by helical X-ray CT, the overall damage distribution along the composite specimen can be monitored in relation to the composite microstructure (e.g., backing fibre bundle cross-over regions, stitching thread cross-over regions, resin-rich regions, fibre misalignment in UD fibre bundles) at different stages of its fatigue life. In the specimen studied, four main damage modes were observed, namely off-axis cracking in the matrix or backing fibre bundles, fibre fractures, sub-surface debonding and longitudinal splitting in the UD layers. Figure 3 shows the extracted fatigue damage within the specimen after 500,000 cycles, where the fibre fractures and longitudinal splits are visualised with respect to the UD and backing fibre bundles (rendered green). The regions with fibre fractures were manually delineated (see red contours in Figure 3d) to highlight the extent of UD fibre fracture damage in 3D. The zinc iodide dye penetrated most of the highlighted fibre fracture region; however, some fibre fractures in region (e) were not stained as they were not connected to the external surfaces. The increased visibility of thin cracks in the high-resolution RoI images confirms the presence of the unstained cracks as shown in Figure 3e and this damage region was also included in the 3D damage visualisation. The effect of staining on damage detectability in this material was discussed in [12]. In addition, its effect on the observed damage development in this material system has been assessed by comparing the damage status of S1 and S2 after 2 million cycles. For S1, the fatigue test was interrupted at 0.5 million, 1.5 million and 2 million cycles, for repeated staining and imaging at each step; while for S2 the fatigue test was interrupted only after 2 million cycles for staining and imaging. The same damage mechanisms, as observed in S3 here, have been seen in S1 and S2. Moreover, the severity of damage in S1 and S2 is on the same level, with the occurrence of a few small regions of UD fibre fractures near backing fibre bundle cross-over regions. This indicates that there is no obvious influence of staining on the observed damage scenario. The evolution, distribution and interaction of the observed damage mechanisms in S3 will be presented and discussed in the following sections.

### 3.1. Off-Axis Matrix and Interface Cracks

It has been challenging to observe off-axis cracks in this material by X-ray CT due to the closing of those cracks to below the resolution limit after load removal [15]. With the aid of contrast agent, we are able to observe the development of off-axis matrix cracks from the specimen edges and also off-axis cracks between backing fibres.

**Figure 4:**
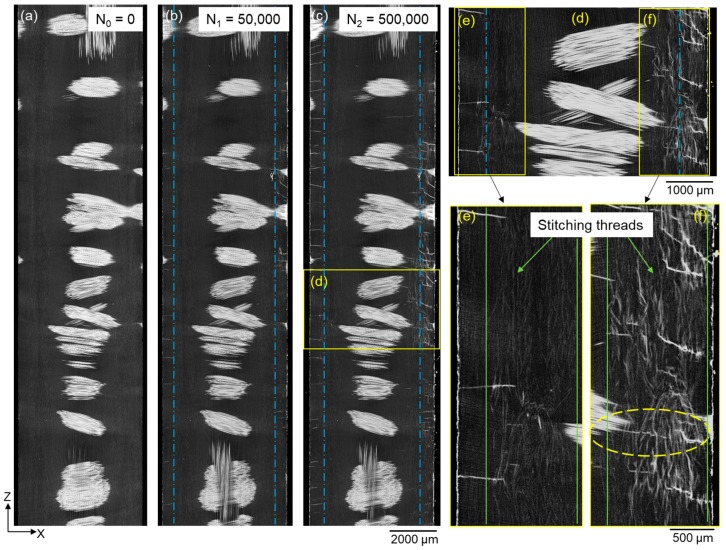
X-ray CT virtual XZ-1 section (position indicated in Figure 3c) of the sample after (**a**) 0, (**b**) 50,000 and (**c**) 500,000 cycles showing the evolution of off-axis matrix cracks initiated from specimen edges. (**d**) Magnified view of the highlighted region in (**c**) showing the off-axis matrix cracks in relation to stitching threads. (**e**,**f**) Magnified views of the highlighted regions in (**d**) showing the polymer stitching threads (light grey) and stained debonding along stitching threads (white). Dashed blue lines indicate the front of off-axis cracks and solid green lines mark the column where the stitching threads are located.

Stained off-axis cracks in the matrix were found to have initiated from specimen edges after 50,000 cycles (N_1_) as a result of the axial tensile stress (see Figure 4b). After exposure to more fatigue cycles (N_2_), the crack density increases dramatically. The observed increase in off-axis cracking has also been reported by Jespersen et al. [15] in a similar material. It is difficult to see the polymer stitching threads as their X-ray absorption capability is similar to that of the matrix material, as can be seen in the magnified views in Figure 4e,f. It has been observed in Figure 4d,f that the off-axis cracks in the matrix tend to be deflected when propagating into the stitching threads. This highlights the effect of stitching threads on delaying the propagation of off-axis matrix cracks from the edges by debonding. Once a crack crosses the column of material containing the stitching threads, it joins the off-axis cracks between backing fibres, as shown in the highlighted ellipse in Figure 4f.

**Figure 5:**
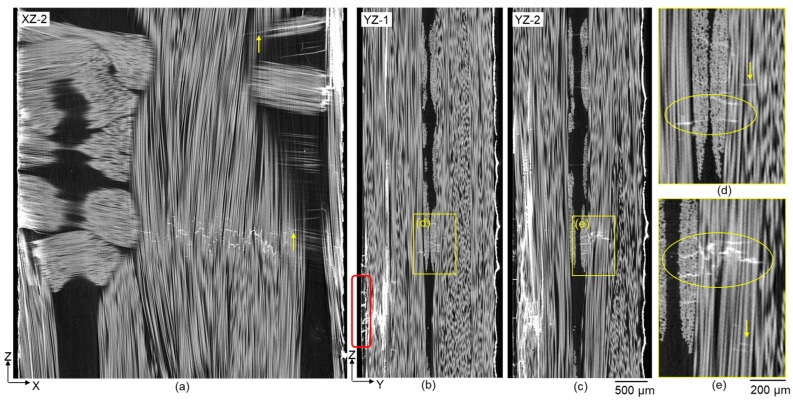
X-ray CT virtual (**a**) XZ-2 and (**b**) YZ-1 and (**c**) YZ-2 sections (positions indicated in Figure 3b,c) recorded after 500,000 cycles showing crack paths connecting off-axis cracks in the backing fibre bundles and fibre fractures in the UD fibre bundles. (**d**,**e**) Magnfied views of the highlighted regions in (**b**,**c**) showing the connected off-axis cracks (marked with ellipses) and also isolated UD fibre fractures (marked with downward-arrows).

The presence of off-axis cracks in between backing fibres has been reported in a number of fatigue studies on this material [5,15] and is observed here after 500,000 cycles (see Figure 5). Only off-axis cracks that are stained were detectable with the current CT acquisition set-up. These stained off-axis cracks are distributed at different heights in the specimen and multiple ones sometimes occur within a single backing fibre bundle; these tend to be distributed vertically relative to one another due to stress shielding [16]. The regions denoted by the upward arrows in Figure 5a and the highlighted ellipses in the YZ sections shown in Figure 5d,e highlight the joining of stained off-axis matrix cracks in the backing layer and stained fibre fractures in the UD bundles. This confirms the presence of a connecting path between the two damage modes. This observation supports the hypothesis [5] that off-axis cracking in backing fibre bundles triggers fibre fractures in the adjacent UD fibre bundles. It is worth noting that the stained fibre fractures were not necessarily directly connected to the off-axis cracks in some cases, as highlighted by the downward-arrows in Figure 5d,e. However, the fact that these fibre fractures were stained successfully means that they were connected to the free surface, either through cracks running into the specimen surfaces, or tunnel-cracks (through backing bundles) extending to the edges.

### 3.2. Fibre Fractures in UD Fibre Bundles

The nucleation of UD fibre fractures was found to correlate with two micro-structural features; regions where backing fibre bundles cross-over (near mid-thickness of the specimen, see Figure 3a–c) and regions where stitching threads cross-over (near the specimen surface, see the red boxes in Figure 5b and Figure 8c). The development and distribution of UD fibre fractures from these two regions were tracked in the time-lapse CT images, and the results are presented and discussed in this section. 

**Figure 6:**
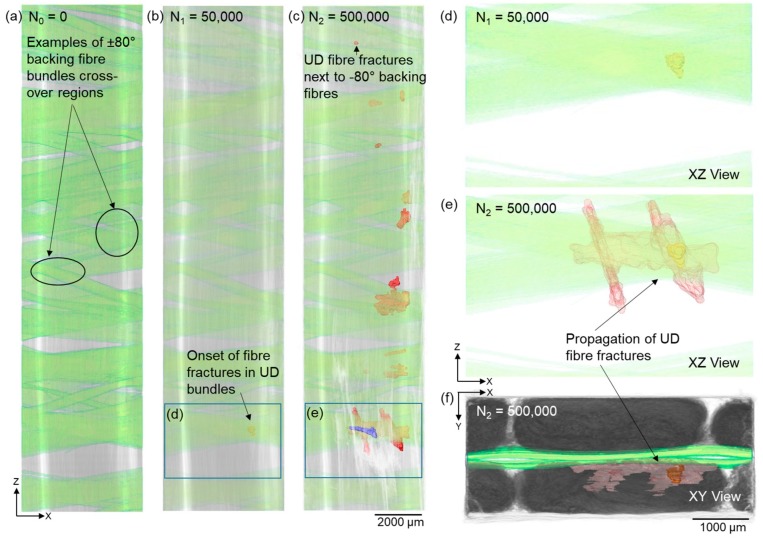
X-ray CT volume rendering of the backing fibre bundles (**a**) after 0 cycles showing the cross-over of the ±80° backing fibre bundles, as well as UD fibre fractures next to backing fibre bundles after (**b**) 50,000 and (**c**) 500,000 cycles, together with magnified views in (**d**,**e**) and in (**f**) the evolution and distribution of UD fibre fractures initiated from backing bundles after 500,000 cycles (refer to Figure 3 for explanations of colour coding). For 3D visualisation see the supplementary video.

As mentioned above, the regions of UD fibre fractures were manually delineated (see Figure 3d) to help visualise the 3D morphology of this damage mode. Fibre fractures were observed in the UD fibres adjacent to the cross-over regions of backing fibre bundles after 50,000 cycles, which is consistent with the observations of previous studies [5,8]. The onset of UD fibre fracture appears to be localised next to backing bundle cross-over regions (see Figure 6b,d). After 500,000 cycles, seven fibre fracture regions were observed on the right-hand side of the specimen width; these were approximately evenly distributed (~2–3 mm) along the 20 mm length. Out of the seven damage regions, six were next to the backing bundle cross-over regions, while one small region was located next to −80° backing fibres only (see Figure 6c). As can be seen in Figure 3c, it should be noted that most of the UD fibre fractures (rendered red) developed near the UD fibre fracture site (rendered blue) developing on the other side of the backing layer. This site is at the same height (in Z) as the site of damage initiation of fibre fractures after 50,000 cycles (see Figure 6b–e). This is the location where UD fibre fractures propagated most widely within the specimen after 500,000 cycles. 

**Figure 7:**
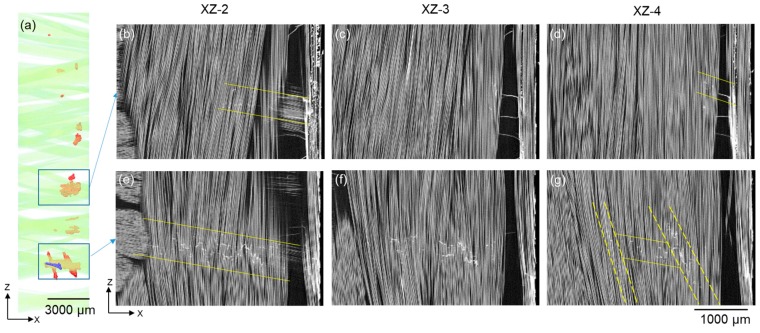
(**a**) XZ view of the rendered UD fibre fracture damage near the backing bundles after 500,000 cycles. (**b**–**g**) X-ray CT virtual XZ sections illustrating the distribution of UD fibre fractures at different positions through thickness in the specimen. The Y-positions of the XZ sections are shown in Figure 3c.

Figure 6e,f shows the propagation of fibre fractures (shown red) between 50,000 and 500,000 cycles. The propagation here was seen to be mostly along the length and width directions rather than through thickness. In the case with backing fibres oriented at ±45°/90° [8] fatigued at maximum strain of 1%, fibre fracture was observed across the full width of the cross-over region and then propagated in the thickness direction. This difference in propagation sequence indicates the necessity of choosing the appropriate arrangement of backing fibres in the composite design for different wind turbine blade requirements. As can be seen in Figure 6e, this damage region has two elongated branches. A local variation in fibre orientation is evident in the 0° fibre bundle (see Figure 7g), which could be the cause of this damage morphology. In other words, 0° fibre alignment could be important in controlling the propagation of UD fibre fractures.

The distribution of UD fibre fractures in relation to the local microstructure within one damage region is revealed by the raw X-ray CT images where individual fibre fractures can be seen (see Figure 7). In the XZ sections near the backing fibre bundles, the distribution of fibre fractures tends to be aligned with the backing fibre bundle in contact with the UD fibres, as shown in the XZ-2 sections. This is also true for the UD fibre fractures at the edge of the UD bundle (see Figure 7a). Further away from the backing bundles, the distribution of fibre fractures is less dependent on backing fibre orientation but more affected by local fibre orientation in the UD bundle, as in the case for the XZ-3 and XZ-4 sections in Figure 7. It is noteworthy that the UD fibre fractures are seldom aligned in a continuous line; instead they tend to cluster in small numbers at different heights.

**Figure 8:**
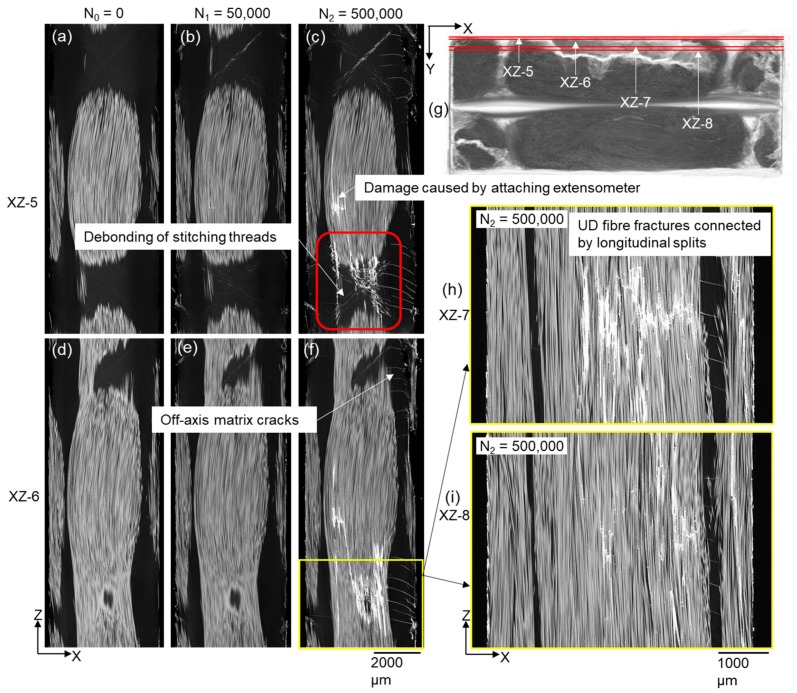
(**a**–**f**) and (**h**,**i**) Sub-surface X-ray CT virtual XZ sections showing the development of UD fibre fractures from the debonding of stitching threads. The Y-positions of the XZ slices are shown in (**g**).

UD fibre fractures were also observed to occur near one specimen surface after 500,000 cycles (see Figure 3a and Figure 5). Figure 8 shows sub-surface XZ sections in this region through the fatigue cycling. It is evident that sub-surface debonding of stitching threads (see Figure 8b,c) is associated with fracture of the adjacent UD fibres in this region, presumably because of higher local stresses. It is worth noting that a number of longitudinal splits developed, connecting the UD fibre fractures that were relatively more distanced from each other (see Figure 8h). As shown in Figure 3a, this damage site is located on the opposite side of the backing fibre bundles to that where fibre fractures initiate near the cross-overs after 50,000 cycles; this means that the damage sites are in two UD fibre bundles. However, these two damage sites are at a similar height, and the extents of propagation through the bundle thickness are similar. It is worth mentioning that another damage region was found further above the debonded stitching threads, which was caused by attaching the extensometer during the fatigue test. 

### 3.3. Sub-Surface Debonding of Stitching Threads and Longitudinal Splitting

**Figure 9:**
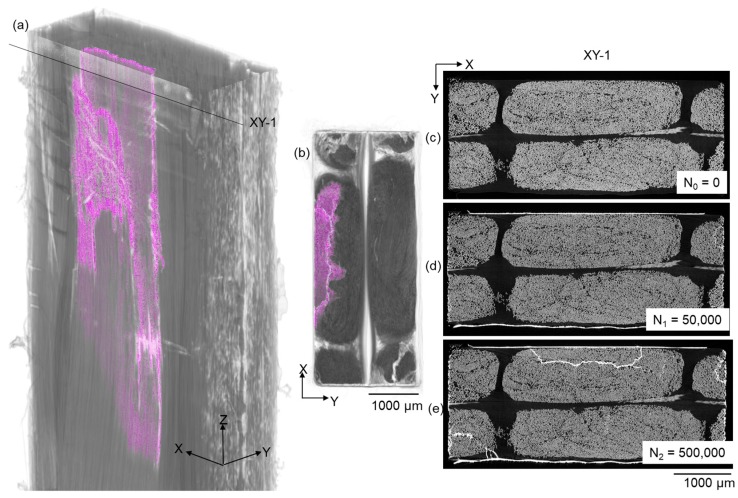
(**a**) Perspective and (**b**) plan view of the X-ray CT 3D volume rendering showing the extent of longitudinal splitting (purple), and an X-ray CT virtual XY section (position indicated in (**a**)) after (**c**) 0, (**d**) 50,000 and (**e**) 500,000 cycles showing the evolution of longitudinal splits.

Extensive longitudinal splitting has been observed in the UD bundle where sub-surface debonding of stitching threads at the cross-over region of the threads has occurred. The longitudinal splits in the UD fibre bundle are extracted and visualised in Figure 9. Longitudinal splitting tends to initially occur in the edge UD bundles due to the edge effect (see Figure 9d). After 500,000 cycles, a longitudinal split originated from a sub-surface debonded region (see Figure 5a and Figure 9e). A section of the UD fibre bundle is separated from the full bundle as shown in the XY-1 plane after 500,000 cycles, and also in the 3D view in Figure 9a. Apart from the fact that these splits are closely correlated with the UD fibre fractures, the splits lower the lateral constraint of the originally closely packed UD fibres, which could potentially make the composite susceptible to compressive loads and moisture. It could be inferred that if the wind turbine blades experience bending fatigue, longitudinal splitting could be a detrimental damage mode. 

## 4. Conclusions

In this paper, a time-lapse ex-situ helical X-ray CT imaging strategy assisted by staining was used to track the development of fatigue damage under tension-tension fatigue in a non-crimp UD GFRP. In essence, the contrast agent could favour the observation of damage where a penetration path exists, but this could be both the weakness and the strength of this method. The weakness is that a damaged region without connection to the outer surface could not be stained; while the strength is that we are able to identify the connection of different damage modes based on the stained path, even for cases where the full crack path is difficult to be ascertained using X-ray CT. This enables us to experimentally prove the hypothesis on the linking of the different fatigue damage mechanisms and also provide insights into their interaction in 3D. Helical X-ray CT makes it experimentally feasible to follow the fatigue damage evolution over a sufficiently long region in the composite along the UD fibre direction. Overall, four main damage modes were identified,

- off-axis matrix and interface cracking,
- UD fibre fracture,
- sub-surface debonding of stitching threads,
- longitudinal splitting.

Off-axis matrix cracks initiating from the specimen edges were sometimes deflected by stitching threads by debonding. Off-axis cracks between backing fibres were found to be associated with UD fibre fractures evidenced by the penetration path of the contrast agent. Moreover, these UD fibre fractures tend to nucleate and propagate locally in the vicinity of cross-over regions of backing bundles instead of being evenly distributed along the UD fibre direction. In addition, UD fibre fractures also tend to be initiated by the presence of extensive debonding and longitudinal splitting, which were found to develop from debonding of the stitching threads near surface. The isolation of the UD fibre bundle caused by longitudinal splitting potentially makes the composite susceptible to compression and bending loads as well as environmental impact in service. It could be inferred from the results here that further research into the better design of the positioning stitching threads, and backing fibre cross-over regions is required in the future, as well as new approaches to fix the positions of UD fibres. The work presented here (all the X-ray CT datasets are available online [17]) could be of significance to the further improvement of analytical and numerical models to predict the fatigue failure of composite materials.

## Supplementary Materials

The following are available online at http://www.mdpi.com/1996-1944/11/11/2340/s1, Video S1: A supplementary video accompanies Figure 6.
